# Induction of Neurite Outgrowth in PC12 Cells Treated with Temperature-Controlled Repeated Thermal Stimulation

**DOI:** 10.1371/journal.pone.0124024

**Published:** 2015-04-16

**Authors:** Tada-aki Kudo, Hiroyasu Kanetaka, Kentaro Mochizuki, Kanako Tominami, Shoko Nunome, Genji Abe, Hiroyuki Kosukegawa, Toshihiko Abe, Hitoshi Mori, Kazumi Mori, Toshiyuki Takagi, Shin-ichi Izumi

**Affiliations:** 1 Division of Oral Physiology, Tohoku University Graduate School of Dentistry, Sendai city, Miyagi, Japan; 2 Liaison Center for Innovative Dentistry, Tohoku University Graduate School of Dentistry, Sendai city, Miyagi, Japan; 3 Tohoku University Graduate School of Biomedical Engineering, Sendai city, Miyagi, Japan; 4 Cell Resource Center for Biomedical Research, Institute of Development, Aging and Cancer, Tohoku University, Sendai city, Miyagi, Japan; 5 Division of Oral Dysfunction Science, Tohoku University Graduate School of Dentistry, Sendai city, Miyagi, Japan; 6 Department of Physical Medicine and Rehabilitation, Tohoku University Graduate School of Medicine, Sendai city, Miyagi, Japan; 7 Institute of Fluid Science, Tohoku University, Sendai city, Miyagi, Japan; 8 IFG Co., Ltd, Sendai city, Miyagi, Japan; Institute of Biochemistry and Biotechnology, TAIWAN

## Abstract

To promote the functional restoration of the nervous system following injury, it is necessary to provide optimal extracellular signals that can induce neuronal regenerative activities, particularly neurite formation. This study aimed to examine the regulation of neuritogenesis by temperature-controlled repeated thermal stimulation (TRTS) in rat PC12 pheochromocytoma cells, which can be induced by neurotrophic factors to differentiate into neuron-like cells with elongated neurites. A heating plate was used to apply thermal stimulation, and the correlation of culture medium temperature with varying surface temperature of the heating plate was monitored. Plated PC12 cells were exposed to TRTS at two different temperatures via heating plate (preset surface temperature of the heating plate, 39.5°C or 42°C) in growth or differentiating medium for up to 18 h per day. We then measured the extent of growth, neuritogenesis, or acetylcholine esterase (AChE) activity (a neuronal marker). To analyze the mechanisms underlying the effects of TRTS on these cells, we examined changes in intracellular signaling using the following: tropomyosin-related kinase A inhibitor GW441756; p38 mitogen-activated protein kinase (MAPK) inhibitor SB203580; and MAPK/extracellular signal-regulated kinase (ERK) kinase (MEK) inhibitor U0126 with its inactive analog, U0124, as a control. While a TRTS of 39.5°C did not decrease the growth rate of cells in the cell growth assay, it did increase the number of neurite-bearing PC12 cells and AChE activity without the addition of other neuritogenesis inducers. Furthermore, U0126, and SB203580, but not U0124 and GW441756, considerably inhibited TRTS-induced neuritogenesis. These results suggest that TRTS can induce neuritogenesis and that participation of both the ERK1/2 and p38 MAPK signaling pathways is required for TRTS-dependent neuritogenesis in PC12 cells. Thus, TRTS may be an effective technique for regenerative neuromedicine.

## Introduction

Neurite outgrowth is a key process in the development of functional neuronal circuits and the regeneration of the nervous system following injury. To improve the outcomes of individuals with neurodegenerative diseases and injury, it is necessary to understand and develop optimal extracellular signals that can induce neuronal regenerative activities, particularly those that enhance cellular neurogenesis [1–3].

The rat pheochromocytoma-12 (PC12) cell line is derived from adrenal pheochromocytoma cells (malignant counterpart of chromaffin cells) and represents a well-established model system for investigation of neuronal differentiation and function [4–6]. Treatment with various soluble factors, such as nerve growth factor (NGF) and bone morphogenetic proteins (BMPs), stimulates PC12 cells to differentiate into neuron-like cells [4,7–11]. Specifically, PC12 cells that differentiate following exposure to NGF or NGF-like compounds stop proliferating, show increased acetylcholine esterase (AChE) activity, and become electrically excitable [5,12–14].

Treatment of PC12 cells with NGF induces activation of extracellular signal-regulated kinases 1 and 2 (ERK1/2), which are part of the mitogen-activated protein kinase (MAPK) family, via activation of the NGF receptor tropomyosin-related kinase A (TrkA). Activation of ERK1/2 leads to neurite elongation and development of neuron-like phenotypic characteristics in PC12 cells [15,16]. Differentiation via NGF also requires the participation of p38 MAPK, another MAPK family member, which is mediated by ERK1/2 [17,18].

BMPs, such as BMP2 and BMP4, are members of the large transforming growth factor-β (TGF-β) cytokine superfamily, which mediates various biological events, including neuronal development [19]. BMPs form a complex with two classes of transmembrane receptors, type I and type II [20], and activate two downstream pathways: the TGF-β-associated kinase 1 (TAK1)-p38 MAPK signaling pathway and the Smad signaling pathway [21,22]. BMPs have also been demonstrated to stimulate neurite elongation in PC12 cells and neurons [9,11,23,24]. The neuritogenesis induced by BMPs in PC12 cells is dependent upon BMP-mediated p38 MAPK signaling [25,26].

Thermotherapy, such as magnetic hyperthermia, has been the subject of increasing attention as a safe cancer therapy [27–30]. Additionally, some evidence suggests that a one-time-only transient heat stimulation, such as mild hyperthermia (42.0 to 43.0°C), may protect neurons or neuron-like PC12 cells from neuronal damage [31,32]. However, few studies have examined the individual effect of a mild thermal-cycle-loading [hereafter temperature-controlled repeated thermal stimulation (TRTS)] on neuronal differentiation in these cells.

Therefore, given the possible therapeutic applications of mild TRTS (39.5 and 42.0°C) for inducing neuronal differentiation and regeneration, we examined neuritogenesis and acetylcholine esterase (AChE) activity, which are known differentiation phenotypes of PC12 cells [4,12], following TRTS in PC12 cells. The TRTS used in this study promoted neuritogenesis gradually in PC12 cells without the addition of other neuritogenesis inducers. Here, we report this novel method of regulating neurite initiation and elongation in PC12 cells using TRTS and discuss a possible biological mechanism of TRTS action.

## Materials and Methods

### Cells and reagents

PC12 cells, established by Greene and Tischer [4], were provided by RIKEN BioResource Center (Tsukuba, Japan) through the National Bio-Resource Project of the Ministry of Education, Culture, Sports, Science, and Technology of Japan (MEXT). Recombinant human BMP4 (Peprotech, Rocky Hill, NJ, USA) was dissolved in LF6 buffer solution (5 mM glutamic acid, 5 mM NaCl, 2.5% glycine, 0.5% sucrose, and 0.01% Tween 80). The MAPK/ERK kinase (MEK)1/2-specific inhibitor U0126 (Calbiochem, San Diego, CA, USA) and a negative control for U0126, U0124 (Merck Millipore, Billerica, MA, USA); p38 MAPK-specific inhibitor SB203580 (Enzo Life Sciences, Farmingdale, NY, USA); the TrkA-specific tyrosine kinase inhibitor GW441756 (Axon Medchem BV, Groningen, The Netherlands) were dissolved in diethyl sulfoxide (DMSO; Wako Pure Chemical Industries, Osaka, Japan).

### Analysis of cell growth and neurite outgrowth

PC12 cells were maintained in a growth medium [Dulbecco’s Modified Eagle’s Medium (DMEM)] supplemented with 5% fetal bovine serum (Thermo Fisher Scientific, Waltham, MA, USA), 5% horse serum (Thermo Fisher Scientific), and penicillin/streptomycin at a pre-set temperature of 37°C in a 5% CO_2_ humidified incubator. For the cell growth assay or the neurite outgrowth assay, PC12 cells were seeded in growth medium at 1 × 10^4^ cells per well in 24-well tissue culture plates (TPP, Trasadingen, Switzerland) for the cell growth assay, or in collagen type IV-coated, 24-well culture plates (Corning Incorporated, NY, USA) for the neurite outgrowth assay, and allowed to grow for 24 h. The cells were then cultured in the growth medium continuously for the cell growth assay or placed in the differentiating medium (DMEM supplemented with 1% horse serum and penicillin/streptomycin) for the neurite outgrowth assay. The culture plates were set on heating plates (Thermoplate or Thermoplate II, Tokai Hit, Fujinomiya, Japan), which have the ability to maintain a restrictive surface temperature during TRTS operations. For the TRTS treatment, cultures were heated at two thermal settings (39.5 or 42.0°C of the pre-set surface temperature of the heating plate) for various time periods (total 0–18 h per day) for a maximum of 14 days. For heat-stimulation periods lasting longer than 3 h, a 1-h break was programmed using a PT50DG digital timer (REVEX, Kawaguchi, Japan). Cell number or neuritogenesis was quantified as described previously [9]. In brief, the cells were examined using a phase-contrast microscope (DP72; Olympus, Tokyo, Japan). Four images per well were captured. For the cell growth assay, the attached cell number in the field was counted; and for the neurite outgrowth assay, cells displaying projections 1.5 times longer than the length of the cell body were considered positive. Each data point corresponds to the counts obtained from three or four independent wells.

### Analysis of acetylcholinesterase (AChE) activity

To examine AChE activity (a biochemical marker for neuronal differentiation) in PC12 cells, the choline produced from acetylcholine by endogenous AChE in each sample was quantified using the Amplite Fluorimetric Acetylcholine Esterase Assay Kit (AAT Bioquest, Sunnyvale, CA, USA) with ReadiUse mammalian cell lysis buffer *5X* (AAT Bioquest) in accordance with the manufacturer’s instructions. Assay signals were read with an absorbance microplate reader (GloMax Multi Detection System, Promega, Madison, WI, USA) at 560 nm, which was standardized to the protein concentration in each sample. The concentration of proteins was measured by Bradford’s method with bovine serum albumin as the standard.

### Thermal evaluation of medium during heat stimulation

To measure the temperature of the medium during heat stimulation, we used a temperature sensor (miniature thermocouple) associated with the heating plate (Thermoplate II). The apical end of the sensor was placed into cell-free growth medium (0.5 ml per well) in 24-well culture plates. For temperature equilibration, the 24-well culture plate was set on a heating plate in a 37°C CO_2_ incubator (APC-30D; Astec, Fukuoka, Japan) 24 h before TRTS treatment. Following this, the culture medium on the heating plate was exposed to TRTS (39.5°C or 42°C as pre-set surface temperature of the heating plate) for a total of 18 h, and the thermal change of the culture medium in the plate was monitored every 60 sec. A 1-h break was inserted between 3-h thermal stimulations. The temperature in the medium during TRTS was recorded using a temperature-management software, TEM (Tokai Hit).

### Statistical analysis

The data are presented as the mean ± SD. Significant differences between groups were identified by one-way or two-way analysis of variance followed by Tukey’s test as appropriate. *P*-values < 0.05 were considered statistically significant.

## Results

**Fig 1:**
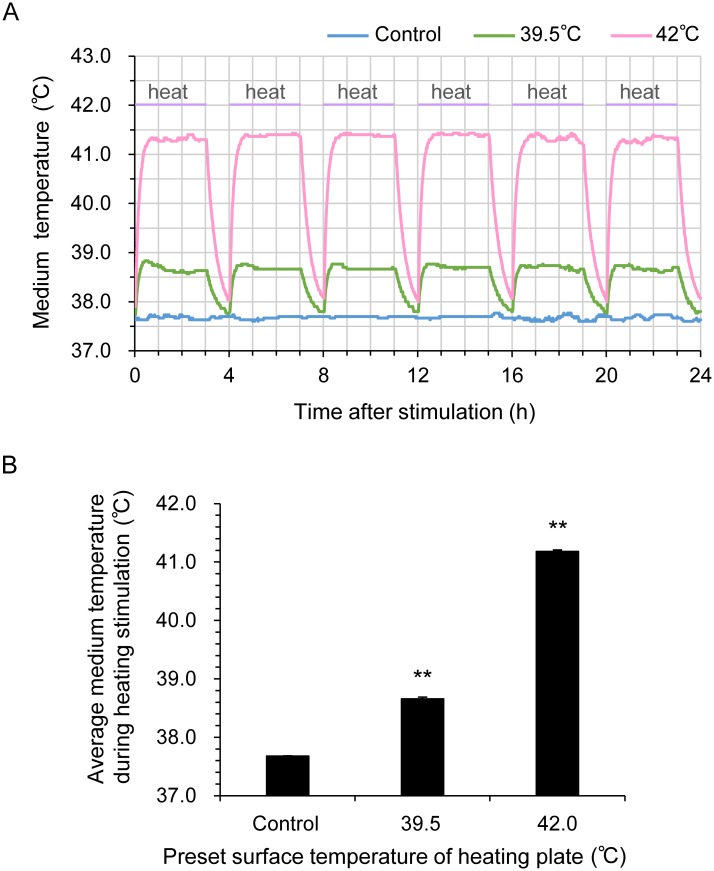
Thermal change of culture medium on a heating plate during TRTS. (A, B) One day before a thermal evaluation, the cell-free growth medium was transferred into a 24-well culture plate. The plate was set on a heating plate in a CO_2_ incubator and incubated for 24 h with a temperature sensor for monitoring medium temperature. (A) The temperatures of the medium during TRTS exposure (for total 18 h) that were recorded every 60 sec are shown. The data represent the mean of three replicates. (B) The average medium temperatures during heating stimulation for 18 h are shown. The data represent the mean ± SD of three replicates. **P < 0.01 vs. no-TRTS controls. Pre-set temperature of the heating plate to warm culture medium was 39.5°C or 42°C. There was a 1-h break between the 3-h thermal stimulations. TRTS, temperature-controlled repeated thermal stimulation.

To assess the effect of TRTS (see Materials and Methods for details) on PC12 cells, we first determined the correlation between the medium temperature in the absence of cells with the surface temperature of the heating plate using two thermal settings (39.5°C or 42°C as pre-set surface temperature). As shown in Fig 1A and 1B, the average culture medium temperature of the wells was adequately maintained at 37.7°C in the absence of TRTS by a temperature-maintaining function of the 37°C CO_2_ incubator for the entire measurement period. Upon TRTS exposure, the average culture medium temperature immediately increased by contact thermal conductance, and the temperature was then maintained at an almost constant level (38.7°C or 41.2°C) for the 3-h continuous thermal stimulation period. Following the 3-h thermal stimulation, the average culture medium temperature rapidly decreased and nearly reached equilibrium temperature every time. These results show that medium temperature was mechanically regulated in a rigorously reproducible manner during TRTS treatment in the present study.

**Fig 2:**
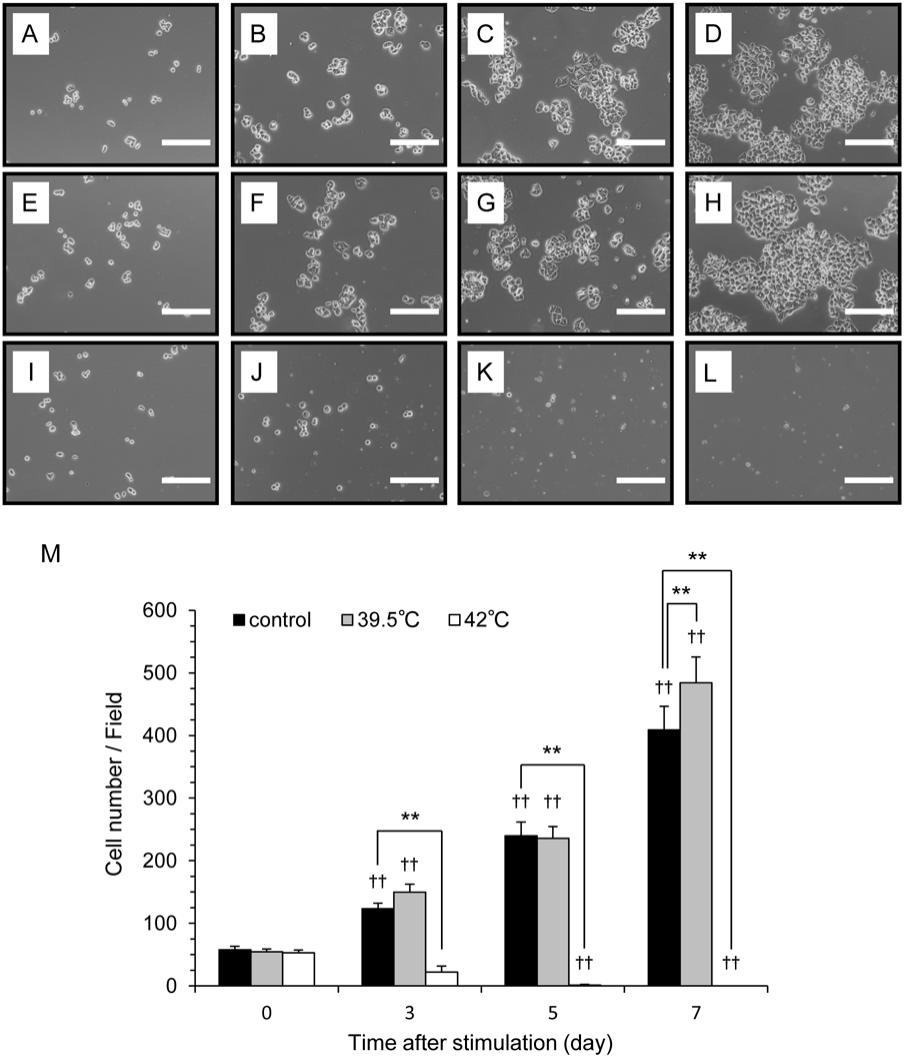
The effect of TRTS on cell proliferation of PC12 cells. (A–L) PC12 cells in cell growth medium were exposed to TRTS at two different temperatures for 18 h/day, or left untreated for 7 days. During TRTS, phase-contrast images were captured on day 0 (A, E, and I), day 3 (B, F, and J), day 5 (C, G, and K), and day 7 (D, H, and L). Phase-contrast images of PC12 cells during incubation without TRTS treatment (A–D); with TRTS at 39.5°C (E-H); or with TRTS at 42.0°C (I-L). Scale bar, 200 μm. (M) PC12 cells in the cell growth medium received TRTS at two different temperatures for 18 h/day, or were left untreated for 7 days, and the number of attached cells on the bottom of the plate was determined. The data represent the mean ± SD of four replicates. ††*P* < 0.01 vs. the day 0 controls with no stimulation. **P < 0.01. TRTS, temperature-controlled repeated thermal stimulation.

To examine the effects of TRTS on the viability of PC12 cells, we next investigated the effect of the two TRTS treatments (pre-set heating plate surface temperature of 39.5°C or 42°C) on cell proliferation in a growth medium. PC12 cells were incubated in growth medium for 7 days and exposed to TRTS for a total of 18 h per day, or left untreated for 7 days, and the number of cells attached to the bottom of the well in each case was evaluated on the indicated days (day 0, 3, 5, and 7). As shown in Fig 2, in response to TRTS treatment at 42°C, PC12 cells significantly decreased in number from day 3 compared to the TRTS-free control cells, and even fewer cells were present on days 5 and 7. In contrast, no significant decrease in cell number was observed between control cells and the cells exposed to TRTS at 39.5°C from days 0 to 7. Rather, TRTS treatment at 39.5°C induced a slight, but significant, increase in cell number on day 7, compared to TRTS-free control cells. These results suggest that TRTS treatment at 39.5°C for a total of 18 h/day is not cytotoxic and has a mild cell-proliferative effect on PC12 cells.

**Fig 3:**
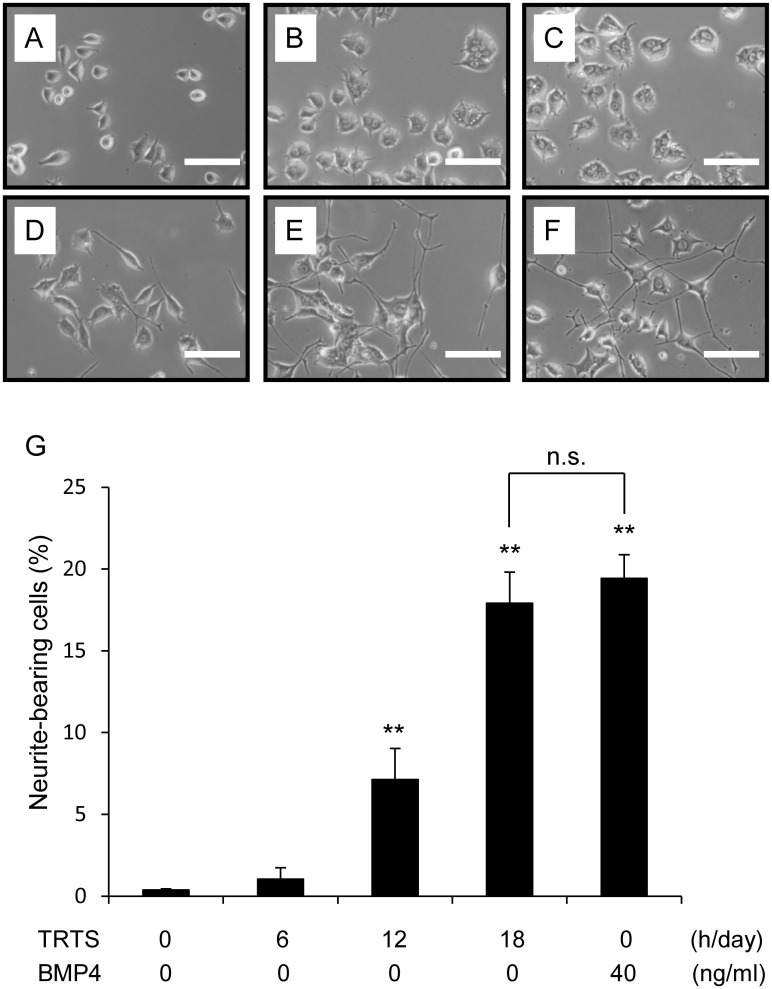
Dose effect of TRTS on neurite outgrowth in PC12 cells. (A–F) PC12 cells in the differentiation medium were treated with 40 ng/ml BMP4, exposed to TRTS at 39.5°C for various time periods, or left untreated for 7 days. (A) Phase-contrast images of PC12 cells on day 0 prior to treatment in cell culture; (B) on day 7 after treatment without stimulation in cell culture; (C) with TRTS for 6 h/day; (D) with TRTS for 12 h/day; (E) with TRTS for 18 h/day; (F) with 40 ng/ml BMP4. Scale bar, 100 μm. (G) PC12 cells were treated with 40 ng/ml BMP4 or TRTS for the indicated time periods, or left untreated, for 7 days, and the percentage of neurite-bearing cells on day 7 was determined. The data represent the mean ± SD of three replicates. **P < 0.01 vs. the day 7 control with no stimulation. n.s., not significant. BMP4, bone morphogenetic protein 4, TRTS, temperature-controlled repeated thermal stimulation.

To examine the effects of TRTS at 39.5°C on the neuronal differentiation of PC12 cells, we next investigated the dose effect of TRTS treatment on neuritogenesis. PC12 cells were either incubated for 7 days in the presence of a positive control (40 ng/ml BMP4) or exposed to TRTS for various time periods (a total of 6, 12, or 18 h per day), and the extent of neurite outgrowth was evaluated. As shown in Fig 3A, prior to TRTS exposure on day 0, the cells were relatively small and round with few visible neurites. Neurite elongation was less than 3% in PC12 cells incubated without stimulation for 7 days (Fig 3B–3G). Treatment with BMP4, an inducer of neurite outgrowth in neurons and PC12 cells [10,23], induced neuritogenesis by day 7 (Fig 3F and 3G). No significant enhancement in neuritogenesis was observed on day 7 in PC12 cells exposed to TRTS for 6 h per day (Fig 3C–3G). In contrast, exposure to TRTS for 12 h or 18 h per day significantly increased the outgrowth of neurite-like projections in PC12 cells in the absence of other neurotropic factors. Moreover, the degree of neuritogenesis observed was similar to that of BMP4 application (40 ng/ml) (Fig 3D, 3E, and 3G), and the morphology of the neurite-like projections generated as a result of TRTS for 18 h per day resembled that of the BMP4-induced neurites (Fig 3E and 3F).

**Fig 4:**
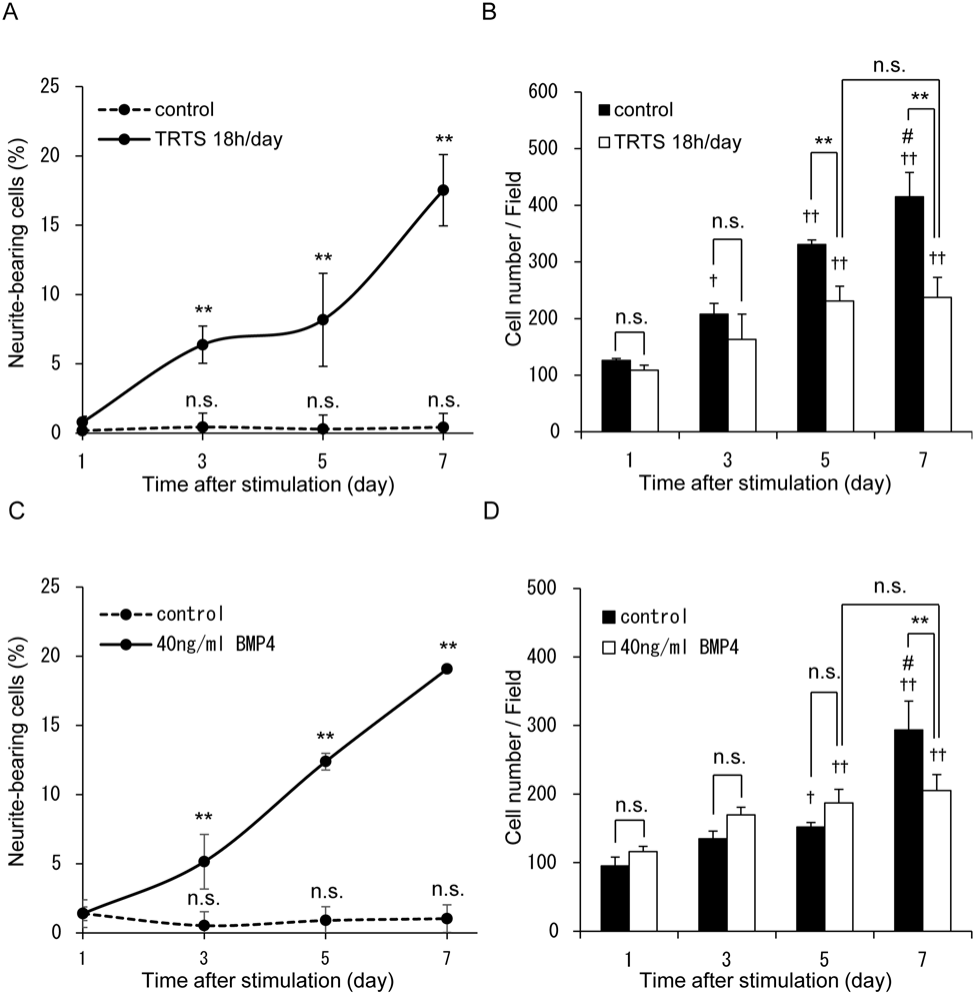
Time course of TRTS-induced neurite outgrowth in PC12 cells. (A) PC12 cells in the differentiation medium were exposed to TRTS at 39.5°C for 18 h/day, or left untreated, for 7 days, and the percentage of neurite-bearing cells on days 1, 3, 5, and 7 was determined. The data represent the mean ± SD of three replicates. **P < 0.01 vs. the day 1 controls with no treatment. (B) PC12 cells in the differentiation medium were exposed to TRTS for 18 h/day, or left untreated, for 7 days and the cell number on days 1, 3, 5, and 7 was determined. The data represent the mean ± SD of three replicates. †*P* < 0.05, ††*P* < 0.01 vs. the day 1 control with no stimulation. **P < 0.01. #*P* < 0.05. vs. day 5 with TRTS. (C) PC12 cells in the differentiation medium were treated with 40 ng/ml BMP4, or left untreated, for 7 days, and the percentage of neurite-bearing cells on days 1, 3, 5, and 7 was determined. The data represent the mean ± SD of the three replicates. **P < 0.01 vs. the day 1 controls with no treatment. (D) PC12 cells in the differentiation medium were treated with 40 ng/ml BMP4, or left untreated, for 7 days and the cell number on days 1, 3, 5, and 7 was determined. The data represent the mean ± SD of three replicates. †*P* < 0.05, ††*P* < 0.01 vs. the day 1 control with no stimulation. **P < 0.01. #*P* < 0.05. vs. day 5 with BMP4. n.s., not significant.

We next evaluated the time course of TRTS-dependent neurite elongation in PC12 cells. We first assessed neuritogenesis and proliferation on days 0–7 in PC12 cells exposed to TRTS at 39.5°C for 18 h per day, or treated with BMP4 (as a control). TRTS-mediated neurite outgrowth occurred gradually in a time-dependent manner (Fig 4A), similar to BMP4-treated neuritogenesis (Fig 4C). In addition, the TRTS-treated PC12 cells also stopped proliferating after day 5, similar to PC12 cells that differentiated in response to BMP4 (Fig 4D), and NGF [5,12,13], but did not exhibit a decrease by day 7 (Fig 4B–4D). These results suggest that TRTS treatment at 39.5°C is not harmful to these cells during neuritogenesis, as are other soluble neurotrophic factors, and that it alone has the potential to induce neuritogenesis in PC12 cells through an unknown and novel mechanism. In fact, TRTS-dependent neurite extension may involve the participation of several intracellular signaling pathways.

**Fig 5:**
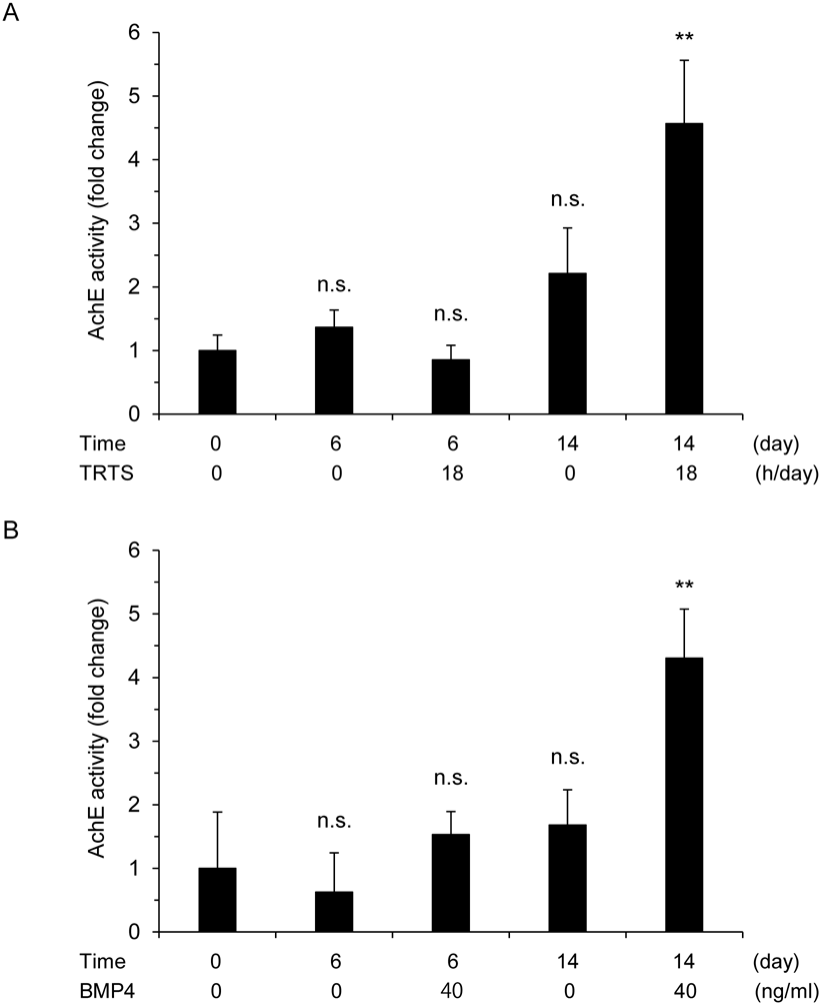
Time course of TRTS-induced AChE activity in PC12 cells. (A, B) Cell lysates from PC12 cells in the differentiation medium were prepared and analyzed for acetylcholine esterase (AChE) activity after treatment with TRTS at 39.5°C (18 h/day) or 40 ng/ml BMP4 for the indicated number of days. The data represent the mean ± SD of three replicates. **P < 0.01 vs. the day 0 control with no stimulation. n.s., not significant vs. the day 0 control with no treatment. BMP4, bone morphogenetic protein 4, TRTS, temperature-controlled repeated thermal stimulation.

We also assessed whether TRTS treatment at 39.5°C increases AChE activity, which is a biochemical marker of neuronal function, in PC12 cells. Cell lysates were analyzed for AChE activity after culture for 6 or 14 days with TRTS treatment at 39.5°C for 18 h per day or in the presence of 40 ng/ml BMP (as a control). As shown in Fig 5, TRTS treatment had no significant effect on AChE activity on day 6. However, similar to BMP4 treatment [10], it significantly increased AChE activity by day 14. These results suggest that TRTS treatment at 39.5°C gradually induces not only morphological differentiation, but also functional differentiation, in PC12 cells.

**Fig 6:**
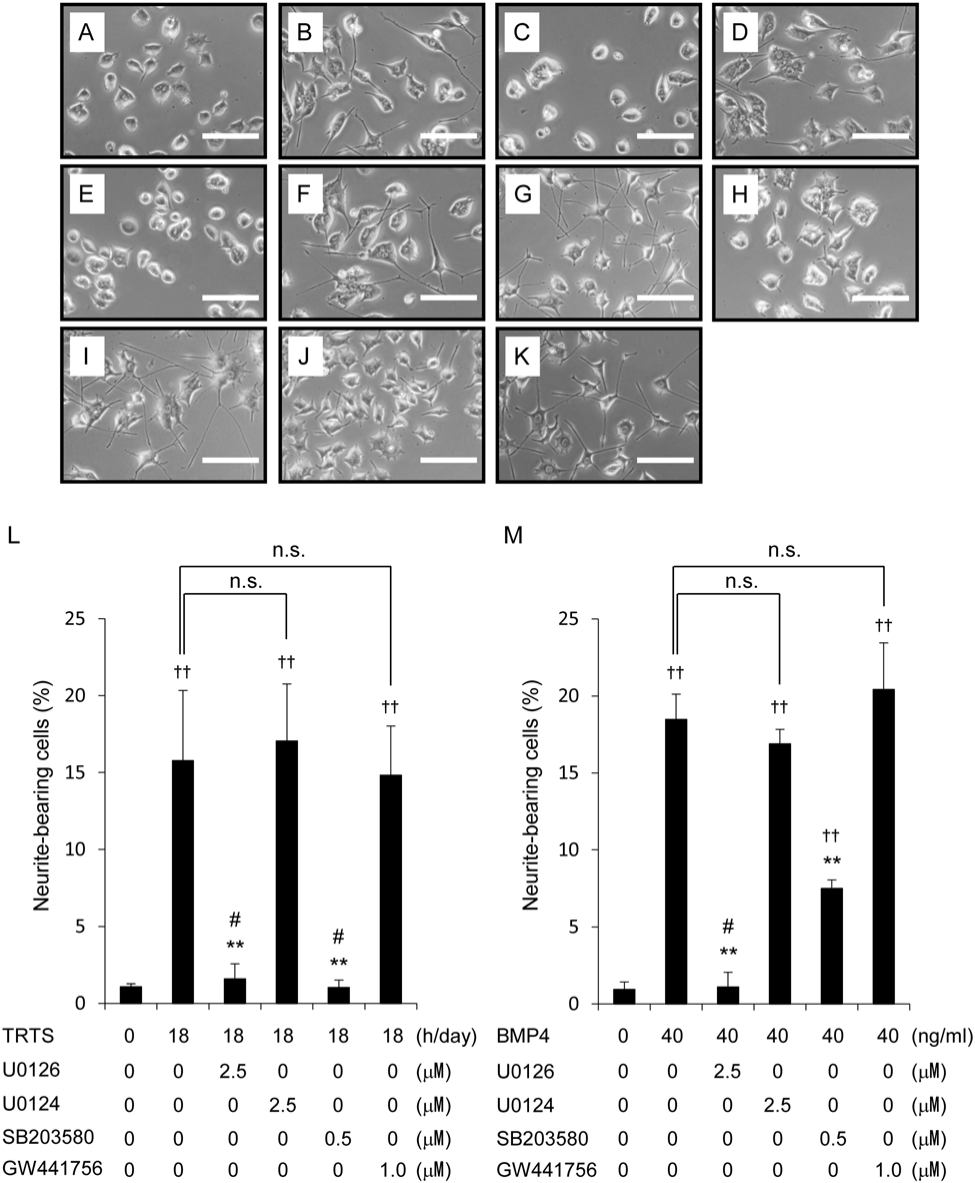
TRTS-induced changes in PC12 cell differentiation after exposure to various inhibitors. (A–K) PC12 cells in the differentiation medium were treated with TRTS at 39.5°C (18 h/day), 40 ng/ml BMP4, or left untreated for 7 days in the presence or absence of 2.5 μM U0126, 2.5 μM U0124, 0.5 μM SB203580, or 1.0 μM GW441756. (A) Representative phase-contrast images of PC12 cells on day 7 after treatment with no stimulation; (B) TRTS (18 h/day); (C) TRTS (18 h/day) plus 2.5 μM U0126; (D) TRTS (18 h/day) plus 2.5 μM U0124; (E) TRTS (18 h/day) plus 0.5 μM SB203580; (F) TRTS (18 h/day) plus 1.0 μM GW441756; (G) 40 ng/ml BMP4; (H) 40 ng/ml BMP4 plus 2.5 μM U0126; (I) 40 ng/ml BMP4 plus 2.5 μM U0124; (J) 40 ng/ml BMP4 plus 0.5 μM SB203580; (K) 40 ng/ml BMP4 plus 1.0 μM GW441756. Scale bar, 100 μm. (L, M) PC12 cells were treated with TRTS at 39.5°C (18 h/day), 40 ng/ml BMP4, or left untreated for 7 days in the presence of the indicated concentrations of U0126, U0124, SB203580, or GW441756. The percentage of neurite-bearing cells on day 7 was assayed. The data represent the mean ± SD of three replicates. ††*P* < 0.01 vs. the day 7 control with no stimulation. **P < 0.01 vs. TRTS or BMP4 alone on day 7. n.s., not significant. #, not significant vs. the day 7 control with no stimulation. TRTS, temperature-controlled repeated thermal stimulation.

Previous work has shown that activation of both the ERK1/2 and the p38 MAPK signaling pathways play a role in neuritogenesis induction in PC12 cells, and that the ERK1/2 and p38 MAPK activation and neurite outgrowth induced by certain neurotrophic factors, such as NGF and BMPs, are blocked by the MEK1/2 inhibitor U0126 or the p38 MAPK inhibitor SB203580 [15,17,18,24, 33,34]. Thus, we next examined whether TRTS-mediated neuritogenesis requires the participation of both the ERK1/2 and the p38 MAPK signaling pathways in PC12 cells. Therefore, prior to the 7-day induction of TRTS-dependent or BMP4-dependent (as a control) neuritogenesis, we pre-treated cells with the MEK1/2 inhibitor U0126 and the p38 MAPK inhibitor SB203580 at concentrations (2.5 μM and 0.5 μM, respectively) previously reported as sufficient to inhibit ERK1/2 and p38 MAPK activation, respectively [10,35,36]. We also scored PC12 cells incubated in the presence of a negative control for U0126 (U0124, 2.5 μM) to assess the inhibition of TRTS-mediated neurite outgrowth. The results presented in Fig 6 show that SB203580 and U0126, but not U0124, suppressed more than half of TRTS- or BMP4-induced neuritogenesis in PC12 cells. These results suggest that TRTS-induced neuritogenesis in PC12 cells requires the participation of both the MEK-ERK1/2 and p38 MAPK signaling pathways.

To further elucidate the signaling pathways involved in TRTS-dependent neuritogenesis in PC12 cells, we next attempted to determine whether TrkA, which is a receptor of NGF and possible upstream signaling molecule in the ERK1/2 and p38 MAPK signaling pathways, is involved. For this purpose, TRTS- or BMP4- mediated neuritogenesis was induced in PC12 cells in the presence or absence of the TrkA kinase-specific inhibitor GW441759. PC12 cells were then scored for neurite outgrowth after a 7-day incubation. As shown in Fig 6F, 6K, 6L, and 6M, GW441576, at a concentration high enough to suppress TrkA activation (1 μM) [37,38], had no significant effect on TRTS- or BMP4-mediated neurite outgrowth. These results suggest that TrkA-derived signaling is not required for TRTS-induced neuritogenesis in PC12 cells.

## Discussion

We report here that neuritogenesis, a key characteristic of neuronal differentiation, can be induced by TRTS at 39.5°C. We also showed that the extent of neurite outgrowth could be dose-dependently regulated by total TRTS-treatment time per day (Fig 3). Similar to induced differentiation via other neurotrophic factors, including BMP4 (Fig 4D), TRTS-mediated induction of neuronal differentiation in PC12 cells halted cell proliferation (Fig 4B). However, this was not accompanied by significant cell death (Figs 3A–3F and 4B–4D), which indicates that TRTS may represent a safe means of promoting nerve regeneration.

Regarding the neuronal differentiation-inducing potential of TRTS at 39.5°C, the percentage of neurite-bearing cells on day 7 in PC12 cells treated with TRTS (18 h/day) was near to and less than 20%, similar to the percentage for 40 ng/ml BMP4-treated PC12 cells (Fig 3B). In contrast, NGF treatment at the normal concentration for neuronal differentiation (50 ng/ml) induces neuritogenesis at least more than 40% on day 7 in PC12 cells [10], suggesting that TRTS, like BMP4, is a milder inducer of neuritogenesis than NGF. In addition to morphological differentiation, TRTS treatment at 39.5°C also up-regulated AChE activity by day 14 in TRTS-treated PC12 cells (Fig 5), similar to BMP4, but not to NGF, which significantly up-regulates AChE activity by day 7 [10]. This suggests that TRTS at 39.5°C could induce functional differentiation, but the pace of functional differentiation induced by TRTS treatment (18 h/day) at 39.5°C is more similar to 40 ng/ml BMP4 treatment than to 50 ng/ml NGF treatment.

In contrast to TRTS at 39.5°C, TRTS treatment at 42°C was markedly cytotoxic to PC12 cells. As shown in Fig 2, after an 18-h TRTS treatment at 42°C in a cell growth assay, almost all of the PC12 cells did not survive for more than a day, implying the significance of fine-tuning thermal conditions when treating cells with TRTS. Future studies should examine the molecular mechanisms that mediate these marked differences in cell response (i.e., cell death and differentiation) between the two TRTS temperatures (39.5°C and 42°C), as well as the receptor(s) sensitive to the heat stimulation.

It is also noteworthy that PC12 cells treated with TRTS at 39.5°C for 12 or 18 h per day for 7 days showed elongated neurites even in the absence of exogenous soluble neurotrophic factors (Figs 3 and 4). In this context, it is possible that TRTS acts synergistically with other neurotrophic factors, including BMP4, in the induction of neuronal differentiation and significantly enhances neuritogenesis induced by those factors in PC12 cells. With regard to the intracellular signaling associated with TRTS treatment for neuronal differentiation in PC12 cells, the present results clearly demonstrate an essential role of the MEK-ERK1/2 and the p38 MAPK signaling pathways in TRTS-dependent neuronal induction in PC12 cells using specific inhibitors for each signaling pathway (U0126 and SB203580, respectively), and a control experiment using BMP4 (as a inducer of neuritogenesis) (Fig 6). Future studies should elucidate whether TRTS treatment immediately induces transient or sustained activation of each pathway, or whether the basal activity of each pathway alone is required for the regulation of TRTS-mediated initiation of neuritogenesis in the cells.

The MEK-ERK1/2 and p38 MAPK signaling pathways that mediated TRTS-induced neuritogenesis in this study are considered to be classic signaling cascades often used by other general neurotrophic factors for stimulating the acquisition of neuronal phenotypes in chromaffin-like (undifferentiated) PC12 cells [6,15,18,39]. This result prompted us to test whether TrkA-mediated signaling is involved in TRTS-mediated neurite outgrowth. To this end, we additionally showed that TrkA tyrosine kinase inhibitor GW441756 did not significantly suppress TRTS-induced neuritogenesis, similar to BMP4-induced neuritogenesis in PC12 cells (Fig 6). This raises the possibility that TRTS-mediated neuritogenesis is independent of TrkA-derived signaling, which is the case for the other neuritogenesis inducers, forskolin and secretin [9,40,41].

In contrast, a previous study suggests that a single 1-h thermal treatment of PC12 cells at 42°C in a water bath (heat shock) may enhance NGF-induced neurite elongation, but that the heat shock-enhancement of neuritogenesis requires MEK-ERK1/2, but not p38 MAPK [3], signaling, implying that the intracellular mechanisms that mediate the heat shock-enhancement of NGF-induced neuritogenesis differ from those that mediate TRTS-induced neuritogenesis.

There is accumulating evidence for membrane and cytosolic proteins that are functionally affected by environmental thermal conditions. Among them, certain heat shock proteins (HSPs) (HSP27, HSP70, and HSP90) are known to directly bind tubulin and thereby regulate microtubule polymerization during neuronal differentiation [42–44]. In addition to HSPs, the transient receptor potential (TRP) channel family members TRPV4, TRPC1, TRPC5, and a related protein, stromal interaction molecule 1 (STIM1, a calcium sensor in the endoplasmic reticulum that regulates the Orai1 plasma membrane channel), are reportedly activated (or inactivated) by temperature changes and involved in the regulation of neuronal differentiation [45–47]. Therefore, the future functional analysis of the relationship between TRTS treatment and these thermo-sensitive proteins mentioned above may help to elucidate the molecular mechanism of TRTS-mediated neuritogenesis in PC12 cells.

In conclusion, we found and characterized a novel effect of TRTS at two different temperatures on the proliferation and differentiation of PC12 cells. Specifically, a non-cytotoxic TRTS treatment can induce neuritogenesis without supplementation of other neurotrophic factors, and requires participation of MEK-ERK1/2 and p38 MAPK signaling, similar to BMP4. Further research into the mechanisms underlying TRTS action and the identification of its critical target signaling molecule(s) leading to neuritogenesis in PC12 cells will be highly valuable and promote the future application of thermal therapy using precisely programmed mild thermal stimulations, like TRTS, towards differentiation therapy and regenerative medicine.
